# Biosensors—Recent Advances and Future Challenges

**DOI:** 10.3390/s20226645

**Published:** 2020-11-20

**Authors:** Paolo Bollella, Evgeny Katz

**Affiliations:** Department of Chemistry and Biomolecular Science, Clarkson University, Potsdam, NY 13699-5810, USA

Biosensors are analytical devices that are able to convert a biological response into an electrical signal. The “golden” biosensor must be highly specific, independent of physical parameters (e.g., pH, temperature, etc.), and should be reusable. The research within the biosensing field requires a multidisciplinary approach that involves different branches of science such as chemistry, biology, and engineering. Biosensors can be categorized based on the biorecognition mechanism: with the biocatalytic group comprising enzymes, the bio-affinity group including antibodies and nucleic acids, and the microbe-based group containing microorganisms. The present Special Issue aimed at summarizing the most recent findings and future challenges regarding biosensors.

In the last six decades, several biosensors have been reported as end-user and time-saving analytical methods for the detection of multiple analytes (e.g., food, clinical, and environmental analytes). In 1962, Professor Leland C. Clark published the first example of an enzyme electrochemical biosensor by entrapping glucose oxidase in a dialysis membrane over a Clark-type oxygen electrode [1]. Moreover, Guilbault and Montalvo reported on glass electrodes coupled with urease to measure urea concentration by means of potentiometry [2]. Besides these first examples, electrochemical transducers have been combined with enzymes, antibodies, and DNA as biochemical recognition components. Nowadays, they represent the largest category of biosensors for food, clinical, and environmental sensing.

The increasing number of scientific publications focusing on biosensors indicates growing interest in the broader scientific community (Figure 1). The present collection of the papers is devoted to all aspects of biosensing in a very broad definition, including, but not limited to, biomolecular composition used in biosensors (e.g., biocatalytic enzymes, DNAzymes, abiotic nanospecies with biocatalytic features, bioreceptors, DNA/RNA, aptasensors, etc.), physical signal transduction mechanisms (e.g., electrochemical, optical, magnetic, etc.), engineering of different biosensing platforms, operation of biosensors in vitro and in vivo (implantable or wearable devices), self-powered biosensors, etc. The biosensors can be represented with analogue devices measuring concentrations of analytes and binary devices operating in the YES/NO format, possibly with logical processing of input signals. 

In this collection, we combined twenty outstanding contributions focusing on different aspects of the biosensing field, mostly highlighting recent advances and future challenges of DNA detection, immunosensing, in vivo electrochemical biosensors, redox enzyme-modified electrode surfaces, photoelectrochemical processes, field-effect transistor-based biosensors, etc., which can be considered as biosensing sub-topics, as reported in Figure 2. A brief summary of each accepted contribution is provided below to encourage the readers to go through them and “visualize” the state of the art within the field of biosensing.

Among the big question marks in biosensing development, Vadgama certainly addressed one of the main challenges regarding continuous and in vivo monitoring in complex media like blood or human tissues. Based on recent findings, electrochemical sensors offer one of the few routes to obtain continuous read-out and implantable devices information referable to specific tissue locations [3]. In this regard, wearable devices are at the forefront in both academic and industrial research on biosensors. The main advantage for wearable technologies is the remote monitoring of human health by biomarkers detection on the skin (e.g., continuous glucose self-monitoring in diabetic patients). Nowadays, the minimally invasive collection of the sample implies the integration of wearable biosensor platforms with microfluidic systems that allow information from the sample to be transmitted directly from the skin to the electrode surface [4,5]. Moreover, the continuous and minimally invasive monitoring of biomarkers has also become of fundamental importance in forensic, biometric, and cybersecurity fields. McGoldrick et al. [6] reported on the possibility of using different bodily fluids for metabolite analysis. This provides an alternative to the use of DNA in order to avoid the backlog that is currently the main issue with DNA analysis by providing worthwhile information about the originator.

Despite the efforts of the scientific community towards the development of minimally invasive and wearable electrochemical biosensors for continuous and in vivo self-monitoring, the fundamental theory behind electrochemical biosensors development still remains a landmark. In particular for enzymes-based biosensors, most bioelectrochemists have focused their attention on possible solutions to tackle direct electron transfer (DET) issues, which are important for enhancing the selectivity and sensitivity of biosensors [7]. Particular attention has been devoted to the case of glucose oxidase (GOx). Despite the huge number of publications on this subject, which unfortunately account for thousands of citations, there is no solid evidence to support DET in GOx, as demonstrated by a stunning statement made by George Wilson: “based on recent experimental results, the observed electrochemical signal corresponds to the FAD cofactor non-covalently bound to the enzyme scaffold that comes out from the redox enzyme upon application of potential, getting adsorbed onto the electrode surface” [8]. 

Beyond the use of GOx as a redox enzyme, there are several enzymes that are able to transfer electrons according to direct or mediated pathways. In nature, many enzymes are attached or inserted into a cell membrane, having hydrophobic subunits or lipid chains for this purpose. Their reconstitution on electrodes allows them to maintain their natural structural characteristics and enables the optimization of their electrocatalytic properties and stability. In this regard, Alvarez-Malmagro et al. [9] discussed different biomimetic strategies to modify electrode surfaces in order to accommodate membrane-bound enzymes, including the formation of self-assembled monolayers of hydrophobic compounds, lipid bilayers, or liposome deposition.

Besides the “classical” enzymes-based biosensors, in the last two decades, many enzymes have been coupled with semiconductive electrodes containing a light-harvesting material in order to develop photoelectrochemical sensing devices. Del Barrio et al. [10] reported on the integration of nanomaterials, such as quantum dots and titanium oxide (TiO_2_) nanoparticles with redox enzymes (e.g., acetylcholinesterase (AChE), glucose oxidase (GOx), etc.), in order to enhance device sensitivity. Considering the successful results in this specific field, future research trends will certainly involve the investigation of different combinations of semiconductor materials and biomolecules and will also consider the possibility of tuning the wavelength to develop a multi-analyte photoelectrochemical biosensor. In particular, Neumann et al. [11] reported on the possibility of combining artificial and natural heme peroxidases with semiconductive electrodes in order to offer new read-out possibilities for hydrogen peroxide and phenolic compounds detection. 

Moreover, the continuous and renown efforts toward the development of nanomaterial-modified electrodes represent another aspect that has been deeply disclosed in the present collection. In particular, Campuzano et al. [12] covered the topic of the modification of electrode surfaces with antibiofouling reagents, which will eventually prevent the non-specific adsorption of biological species on the electrode surface. This is an important topic, especially considering the research on multiplexed and point-of-care devices as cost-effective and selective multianalyte detection methods. Among all the strategies currently available to develop antibiofouling surfaces, the modification of electrode substrates with different biomaterials, including monolayers, transient polymeric coatings, or multifunctional peptides, is particularly attractive and promising. 

In this collection, the use of structured materials, such as nanoporous metals, graphene, carbon nanotubes, and ordered mesoporous carbon, for biosensing applications has been deeply discussed [13].

Recently, sulfur-containing nanomaterials and their derivatives/composites have been extensively employed for the development of alternative biosensing devices. Li et al. [14] summarized the recent findings and future challenges of employing metallic sulfide nanomaterial-modified electrodes, particularly disclosing their specific properties, namely, nanometric scale, water dispersibility, large specific surface area, excellent catalytic activity, conductivity, biosafety, photoluminescence (PL) quenching abilities, photoactivity, and fascinating optical properties. 

Beyond graphene and graphene-like-2D-nanomaterials (e.g., sulfur-containing nanomaterials etc.), Khan et al. [15] reported on the possibility of exploiting MXenes as 2D-layered nanomaterials that provide unique capabilities for bioanalytical applications. These include high metallic conductivity, large surface area, hydrophilicity, high ion transport properties, low diffusion barrier, biocompatibility, and ease of surface functionalization.

Considering special features of nanomaterials, Stasyuk et al. [16] summarized the recent findings about nanozymes. Nanozymes are defined as nanomaterials with enzyme-biomimicking features (e.g., gold nanoparticles that mimic oxidases activity, etc.). This contribution gives an overview of the classification of the nanozymes, their advantages vs. natural enzymes, and their potential practical applications, devoting particular attention to the different synthesis methods developed so far.

Beyond enzyme-based biosensors, immunosensors are also used for the development of point-of-care devices. In particular, Sharafeldin et al. [17] reviewed the most recent findings on 3D-printed immunosensing devices for cancer detection. In the last few years, 3D-printing platforms have been used to produce complex sensor devices with high resolution.

Moreover, aptasensors and DNA-modified electrodes have also been identified as point-of-care devices that are especially useful for quick diagnostics during pandemic emergencies. Santhanam et al. [18] summarized the most recent findings about DNA/RNA-based biosensors, especially considering classical detection method pitfalls, such as for reverse transcription PCR (RT-PCR) and real-time PCR (qPCR), which are considered time-consuming and require specialized professionals and instrumentation. 

On this specific topic, researchers are not focused only on the development of new detection platforms, but they are also addressing potential issues about biosensor sensitivity through different signal amplification methods. Smith et al. [19] reported on recent findings and future challenges surrounding DNA detection based on a direct restriction endonuclease (REase) assay. This assay allows for detection at an attomolar level through an exponential signal amplification method based on a cascade of self-perpetuating restriction endonuclease reactions, which induce continuous cleavage of amplification probes, thus leading to exponential signal amplification. The proposed approach provides a cost-, time-, and labor-effective alternative DNA detection method.

Besides the detection of DNA or antigens, immunosensors, DNA/RNA biosensors, and aptasensors are currently considered in microbiology as powerful tools for the detection of bacteria cells at a single cell level. These biosensors allow for the specific detection of bacteria in complex biological matrices, often in the presence of excessive amounts of other bacterial species [20].

The research within the biosensing field is not only focused on electrochemical and optical transduction techniques but is also currently considering different approaches to obtain a direct electronic read-out, like for electrolyte-insulator-semiconductor (EIS) field-effect sensors, which belong to a new generation of electronic chips. Poghossian and Schöning [21] gave an overview on recent advances and current trends in the research and development of chemical sensors and biosensors based on the capacitive field-effect EIS structure—the simplest field-effect device, which represents a biochemically sensitive capacitor. Similarly, Sedki et al. [22] reported the most recent findings on non-carbon 2D-materials-FET biosensors, discussing how transition metal dichalcogenides (TMDCs), hexagonal boron nitride (h-BN), black phosphorus (BP), and metal oxides impacted the development of the FET-based biosensors.

## Figures and Tables

**Figure 1:**
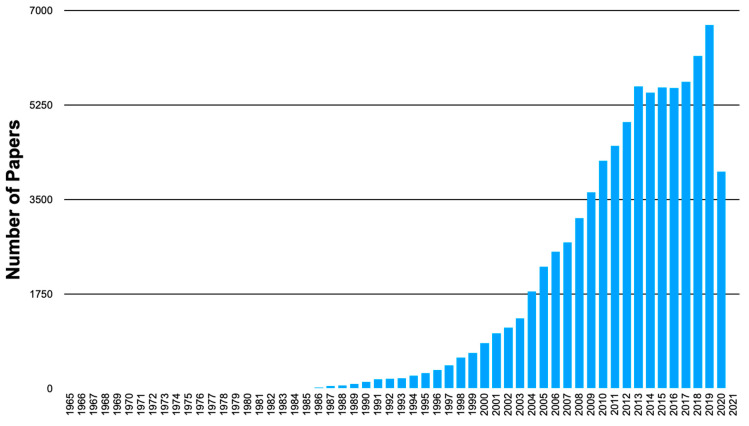
The number of published papers mentioning “biosensors” derived from statistics provided by the Web of Science. The search was performed for the keyword “biosensors” in the topic. (The statistics for 2020 was not complete).

**Figure 2:**
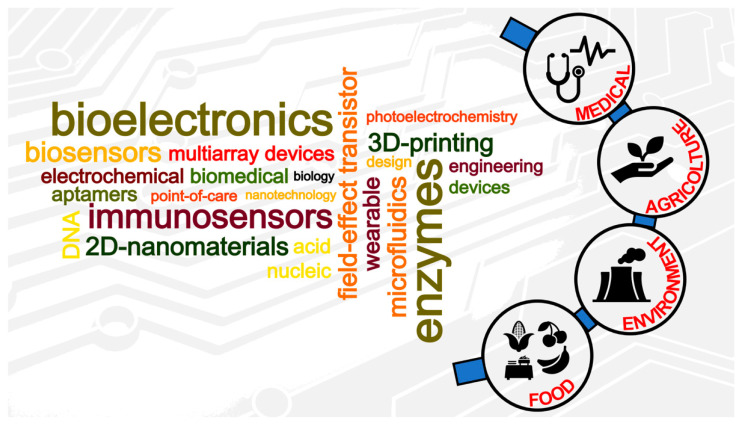
Biosensing topics and sub-topics and possible applications as analytical devices.
